# Exotropia Is the Main Pattern of Childhood Strabismus Surgery in the South of China: A Six-Year Clinical Review

**DOI:** 10.1155/2016/1489537

**Published:** 2016-02-28

**Authors:** Xinping Yu, Zhouduo Ji, Huanyun Yu, Meiping Xu, Jinling Xu

**Affiliations:** ^1^The Eye Hospital of Wenzhou Medical University, Wenzhou 325027, China; ^2^Ophthalmology Department, Zhengzhou Second Hospital, Zhengzhou 450006, China

## Abstract

*Purpose*. To evaluate the distribution pattern and changes of strabismus surgery in children based on the data collected from a local eye hospital in the south of China between 2006 and 2011.* Methods*. A retrospective analysis of all strabismus surgeries in children (<18 years) performed in the Eye Hospital of Wenzhou Medical University between 2006 and 2011 using the integrated information system.* Result*. A total of 2,219 strabismus surgeries were performed during the study period, with concomitant exotropia (44% of all surgeries) more than esotropia (27%, *χ* ^2^ = 42.7, *p* < 0.001). Total number of surgeries increased from 250 in 2006 to 508 in 2011, with a significant increase in concomitant exotropia: 38% of all surgeries in 2006 increased to 47% in 2011 (*χ* ^2^ = 29.4, *p* < 0.001). The increase of intermittent exotropia was approximately 26% of all increments of strabismus surgery between 2006 and 2011.* Conclusion*. Surgery for childhood exotropia was more frequent than esotropia in China. The proportion of exotropia progressively increased, while the proportion of esotropia decreased during these years. Intermittent exotropia was the main increment of strabismus surgery. Further population-based studies are needed to confirm the proportion of surgery and whether the incidence of strabismus surgery increased in China.

## 1. Introduction

Previous epidemiologic studies suggested a relatively higher prevalence of exotropia and a lower prevalence of esotropia in Chinese and other Asian children compared to that of Western children [1–3]. Data based on the clinical reviews in Hong Kong and Singapore supported the same trend [4, 5]. It is also implied from studies that surgery for childhood esotropia was much more common than surgery for exotropia, although the proportion of surgery for esotropia has continued to decline in UK [6–8], Italy [9], and USA [10]. While a study based on an eye center of China showed that surgery for concomitant exotropia was more common than those for esotropia, the amounts of surgery for esotropia also increased from 2003 to 2006. However, the study included patients of all ages who underwent strabismus surgery in the hospital [11]. Whether the pattern distribution of primary surgery for childhood strabismus is significantly different from that of Western populations remains under debate.

The present study aimed to evaluate the pattern distribution and the changes of strabismus surgery in child patients in one region of China based on the data from a local eye hospital.

## 2. Subjects and Methods

This study was approved by the ethics committee of the Eye Hospital of Wenzhou Medical University. All the strabismus surgeries that were performed on patients <18 years of age in our department from 2006 to 2011 were included. All of the data came from the integrated information system of the Eye Hospital of the Wenzhou Medical University. The patients were diagnosed by pediatric ophthalmologist. The following data were recorded for each patient: name, sex, date of birth, age of onset of strabismus, age at the time of surgery, date of strabismus surgery, diagnosis, routine eye examination (including visual acuity and the best corrected visual acuity (BCVA), anterior segment, and refractive and fundus examination), eye movements (EOM), degree of strabismus, stereopsis, sensory fusion, and surgical procedure performed. Strabismus deviation was measured using alternate prism cover tests at 33 cm for nearness and 6 meters for distance. Sensory fusion was tested using Worth 4-dot test and stereoacuity was tested using Titmus stereopsis tests.

The SPSS 19.0 software package was used (SPSS Inc., Chicago, IL, USA). The statistical techniques used were Fisher's exact test (when the total number of observations was <20) and the chi-square test to compare proportions between groups. Independent *t*-test was used to compare the onset age between subjects with some binocular function and those without. The result was regarded as statistically significant when *p* < 0.05.

## 3. Results

### 3.1. Basic Data

In the 6-year period, 2,219 surgeries were performed in our hospital on patients aged 1–17 (9.55 ± 4.31) years, with 54% subjects being male.  Table 1 showed the distribution between sexes and ages for the 6 years. The deviation was 10–145 (47.1 ± 24.6) prism diopters (pd) horizontally and 6–90 (18.2 ± 11.8) pd vertically.  Table 2 showed that 408 cases had myopia (−3.2 ± 3.0 D of spherical equivalent, SE), and 315 cases had moderate hyperopia (3.8 ± 1.7 D of SE) in the right eyes. Both subjects with stereopsis and sensor fusion had older onset age (*t* = 3.82, *p* < 0.001; *t* = 4.35, *p* < 0.001). Better stereo function was also found in subjects with intermittent exotropia (*χ* ^2^ = 39.1,  *p* < 0.001).

### 3.2. Pattern Distribution of Strabismus Surgery

**Figure 1:**
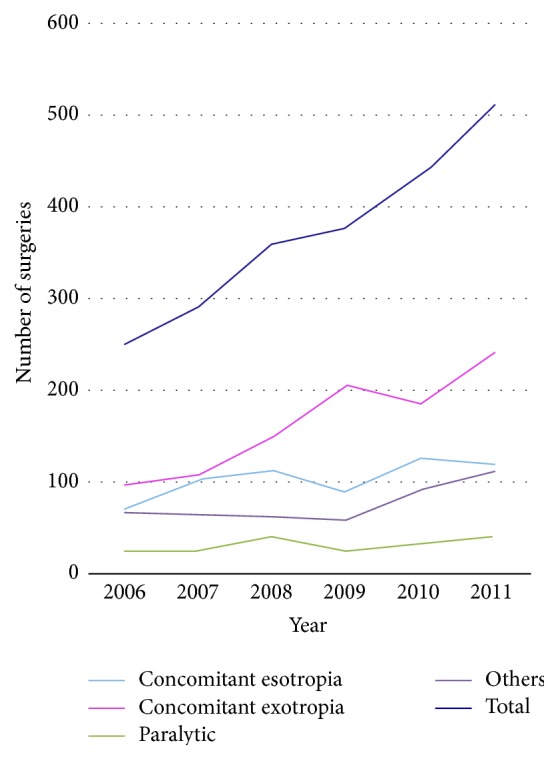
Amounts of the strabismus surgery between 2006 and 2011.

**Figure 2:**
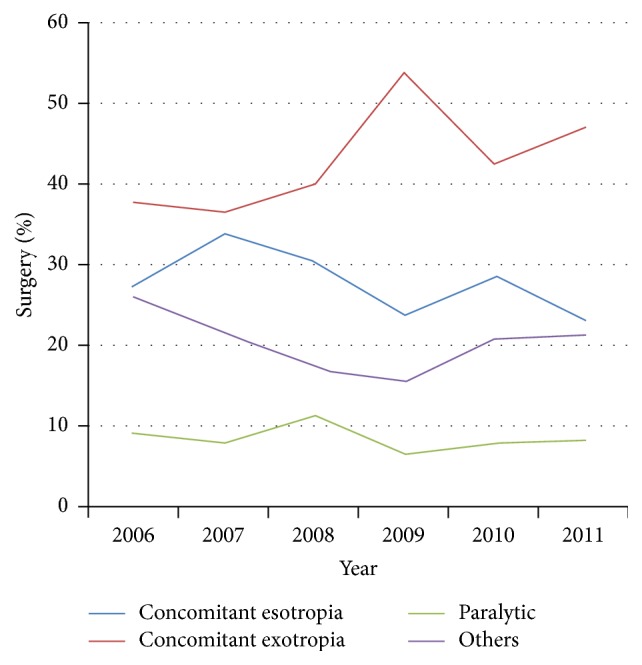
Distribution of surgery from 2006 to 2011.

The number of surgeries for strabismus increased from 250 in 2006 to 508 in 2011. Concomitant exotropia was the most common type of surgery, with a proportion of 44% (973/2219), which was higher than the proportion of surgery for concomitant esotropia (27%, 608/2219, *χ* ^2^ = 42.7, *p* < 0.001) based on the 6-year data. As shown in Figure 1, the amount of surgery for concomitant esotropia has been constant, while the surgery for concomitant exotropia increased between 2006 and 2011.  Figure 2 shows that the proportion of surgery for concomitant exotropia increased from 37.6% in 2006 to 47% in 2011, with the highest proportion of 54% in 2009 (*χ* ^2^ = 29.4,  *p* < 0.001), while the percentage of patients with concomitant esotropia decreased from 27.2% in 2006 to 23.2% in 2011 (*p* = 0.011).

**Figure 3:**
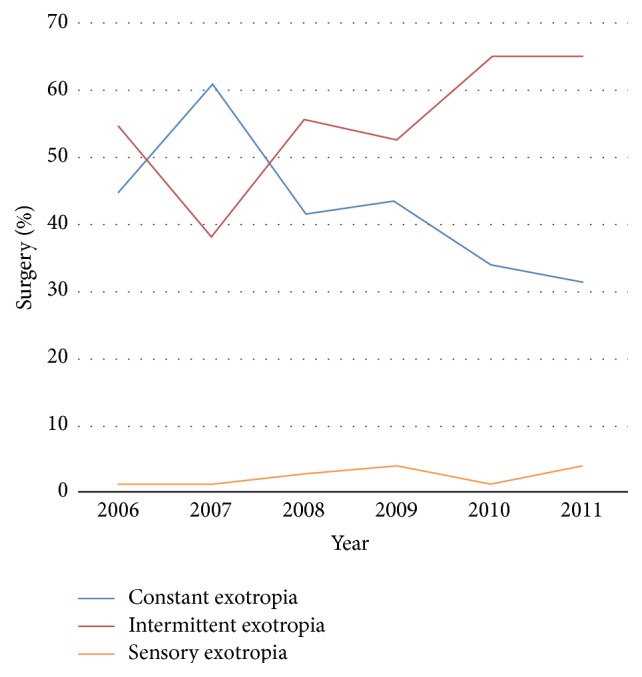
Distribution of concomitant exotropia surgery.

**Figure 4:**
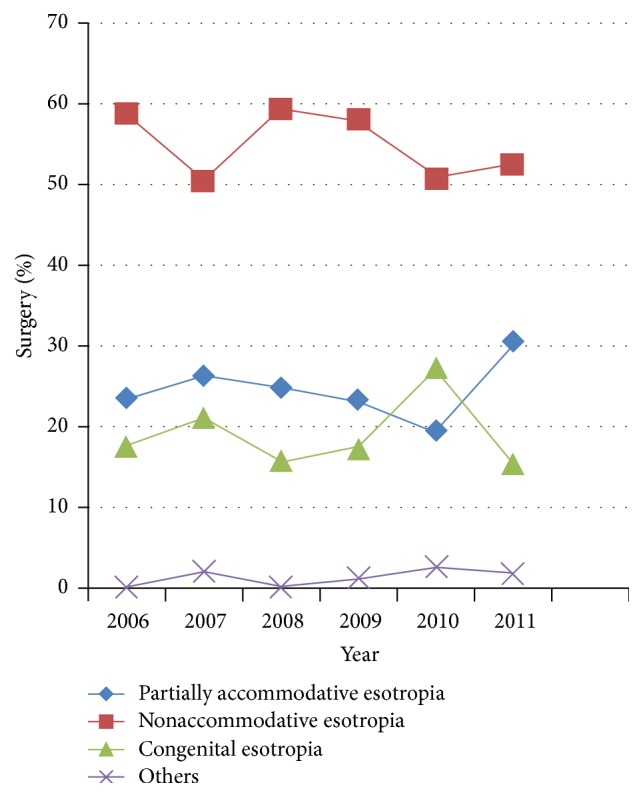
Distribution of the concomitant esotropia surgery.

Figure 3 shows the distribution of the concomitant exotropia surgery between 2006 and 2011, with an increasing trend in the proportion of intermittent exotropia. Intermittent exotropia was the most increased type of strabismus, with increases from 51 (20%) in 2006 to 155 (31%) in 2011 (*p* < 0.001). The increased number of intermittent exotropia surgeries was 501 cases, approximately 26% of the total increments of strabismus surgeries between 2011 and 2006. The distribution of the concomitant esotropia surgery remained essentially the same (Figure 4).

## 4. Discussion

Similar to the results of a Beijing study including patients of all ages [11], we observed that surgery for childhood exotropia was more common than surgery for esotropia based on our hospital data between 2006 and 2011. The proportion of the exotropia surgeries continued to increase, with the amount of surgery for esotropia remaining stable and a decrease in proportion. The increasing number of surgeries for concomitant exotropia, especially for intermittent exotropia, led to an increasing trend of surgeries among children <18 years of age. Strabismus type and onset age were found to be associated with binocular function in the current study, that is, subjects with older onset age and intermittent exotropia.

Our results suggest that more surgeries are performed for exotropia than for esotropia in Chinese patients based on the pattern distribution of surgery, which is the opposite of the pattern observed in Western populations. Weakley et al. found that the most common surgeries in a Western population were for esotropia, although this decreased from 59% (553/930) of primary strabismus surgeries between 1990 and 1994 to 51% (729/1,424) between 2005 and 2009 [10]. It has also been implied that the surgeries for esotropia are much more frequent than surgeries for exotropia in Scotland, Tayside, and England, although the number of surgeries for esotropia continually decreased [6, 8].

Similar to the studies conducted in Western populations [6, 8, 10], the proportion of surgeries for esotropia decreased in our study. This may be explained by earlier detection and a trend of full hypermetropic spectacle correction treatment, which would decrease the demand for a procedure to correct esotropia [12]. We agree with this opinion and noted that the standard optical correction for esotropia was promoted in China during these years: screening of children in preschool and elementary school was promoted during these years, and patients with strabismus were identified and treated earlier with standard optical treatment [13–16]. A study conducted in Britain suggested that preschool screening does not result in a decline in strabismus surgery [6, 17].

The number and proportion of surgeries performed for concomitant exotropia, especially intermittent exotropia, were found to progressively increase in our study. This trend was also reported by Weakley et al., who found that the proportion of surgery for exotropia increased from 25% in 1990 to 49% in 2009 and stated that intermittent exotropia represented an increasing proportion of strabismus referrals [10]. The increase in surgeries for intermittent exotropia may be related to the following: intermittent exotropia was the most frequent and predominate childhood strabismus in Chinese populations [4, 5]. Furthermore, the incidence of childhood intermittent exotropia was suggested to be increased in Chinese children during recent decades, which may be related to the development of myopia [4]. Additionally, the effect of intermittent exotropia on the quality of life (QOL) of the children and their parents was evaluated, which educated them that they have a choice of surgical treatment [18–21]. Finally, although the treatment choice for intermittent exotropia is still under debate, the surgical treatment of intermittent exotropia was proven to be effective: surgery combined with orthoptic therapy was more effective than nonsurgical treatments [22]. In a population-based study, approximately 50% of surgeries succeeded and 45% had a good stereopsis with an average of 8 years of follow-up with surgical treatment [23], while approximately 4% of patients resolved without treatment [24].

Several studies evaluated the main components of surgery for childhood intermittent exotropia: timing and decision to perform surgery [21, 25], whether the exotropia should be initially overcorrected postoperatively [26, 27], whether unilateral surgery or bilateral surgery has better outcomes [28, 29], and whether the patient's quality of life benefits from corrective surgery [29]. As surgery for intermittent exotropia progressively increases due to our study and others [10], further studies are needed to achieve more definitive conclusions.

A limitation of the present study is that it is not a population-based study. However, the Eye Hospital of Wenzhou Medical University is a government funded hospital that undertakes most of the strabismus surgeries for the local population. Referrals to our department include those from general practitioners, general optometrist, and general outpatient departments of district hospitals in Wenzhou region. It should reflect the pattern distribution of strabismus surgery in the Chinese population to a certain extent. Whether it is an increase of strabismus surgeries or a change of pattern distribution in Chinese populations, these variables should be evaluated in a population-based study further.

In summary, there were more surgeries performed for exotropia than for esotropia in China, which may be the opposite of the pattern of surgeries in Western populations. A progressive increase of strabismus surgery, mainly for intermittent exotropia, was observed between 2006 and 2011 in the current study. Further population-based studies are needed to confirm the proportion of surgeries performed and whether the overall incidence of strabismus surgery increased in China.

## Figures and Tables

**Table 1:** Age and sex distribution ($\bar{x}\;\pm \;s$).

	2006	2007	2008	2009	2010	2011
Male	136 (54.4%)	157 (53.6%)	193 (53.8%)	204 (54.1%)	227 (52.5%)	286 (56.3%)
Female	114 (45.6%)	136 (46.4%)	166 (46.2%)	173 (45.9%)	205 (47.5%)	222 (43.7%)
Age	9.53 ± 4.11	9.44 ± 4.42	9.77 ± 4.24	9.75 ± 4.43	9.62 ± 4.40	9.28 ± 4.25
Onset age	3.76 ± 3.58	3.59 ± 3.67	3.84 ± 3.23	3.97 ± 3.42	3.70 ± 3.29	3.44 ± 3.21

**Table 2:** Basic characteristics of the 2219 subjects.

		Number (%)
BCVA	≧0.6 in both eyes	1660 (74.8)
	≦0.3 in one or two eyes	145 (6.5)
	0.3–0.6 in one or both eyes	84 (3.8)
	Uncooperative test	330 (14.9)

Refractive error	SE ≦ −0.5 D myopia	408 (18.3)
	SE ≧ 2.0 D hyperopia	315 (14.2)
	Anisometropia > 1 D	210 (9.7)

Stereoacuity at near	40–480 seconds of arc	496 (22.3)
	>480 seconds of arc	1231 (55.5)
	Uncooperative test	492 (22.2)

Sensory fusion	Normal	181 (8.2)
	Suppression/diplopia	1887 (84.5)
	Uncooperative test	162 (7.3)

EOM	Normal	1478 (66.6)
	Abnormal (overaction/underaction)	741 (33.4)

Surgical procedure	Horizontal	1365 (61.5)
	Vertical/torsional	148 (6.7)
	Horizontal and vertical	706 (31.8)
